# The *domino* SWI2/SNF2 Gene Product Represses Cell Death in *Drosophila melanogaster*

**DOI:** 10.1534/g3.118.200228

**Published:** 2018-05-11

**Authors:** Kaitlyn Ellis, Joanna Wardwell-Ozgo, Kenneth H. Moberg, Barry Yedvobnick

**Affiliations:** * Biology Department, Emory University, Atlanta, Georgia 30322; † Department of Cell Biology, Emory University and Emory University School of Medicine, Atlanta, GA 30322

**Keywords:** Drosophila, *domino*, apoptosis, proliferation, chromatin

## Abstract

The Drosophila *domino* locus encodes DNA-dependent ATPases of the SWI2/SNF2 class. This class of chromatin remodeler is associated with an array of cellular activities encompassing transcription, replication, repair and recombination. Moreover, *domino* was observed initially to maintain a repressive chromatin state via genetic interaction studies with homeotic genes. Although *domino* mutations were also characterized with a cell death phenotype, its association with a death pathway has not been investigated. Here we have used targeted RNA interference to depress *domino* function in the wing. Resultant wing damage phenotypes were found to be enhanced through overexpression of pro-apoptotic loci, and suppressed through loss of function of these loci. Loss of wing margin and blade tissue was correlated with activation of the effector Caspase Dcp-1, a marker for apoptosis. The affected wing regions also exhibited lower levels of the DIAP1 protein, an inhibitor of apoptosis. The lower level of DIAP1 protein was not correlated with an effect on the activity of a DIAP1 gene transgenic reporter (*thread-LacZ*), suggesting that loss of DIAP1 occurred post transcriptionally. In some cases excessive cell proliferation within the targeted tissue, measured through BrdU incorporation, was also observed. Finally, we used a transgenic reporter construct to monitor the chromatin state upstream of the proapoptotic *reaper* locus. In genotypes exhibiting targeted *domino* loss and wing phenotypes, we observed increased reporter activity only in the affected areas. These data support the conclusion that *domino* normally functions to maintain pro-apoptotic genes in a repressed state.

The Drosophila *domino* (*dom*) locus was identified during an enhancer trap screen for *P* element-mediated disruptions of hematopoiesis (Braun *et al.* 1997). Homozygous *dom* larvae were observed to be deficient in hemocytes and exhibited lymph glands that contained necrotic and melanized cells. The substantial cell death of differentiating hemocytes is associated with defective host defense against septic injury, when *dom* is combined with other immune system mutations (Braun *et al.* 1998). Aberrant phenotypes of *dom* mutant larvae were also noted in several other proliferating tissues, including imaginal discs, brain and germline (Braun *et al.* 1997, Ruhf *et al.* 2001). Moreover, mutant clones of strong *dom* alleles are not recovered, even in the genetic background of a *Minute* mutation (Ruhf *et al.* 2001), demonstrating that *dom* function is essential for cell viability. The *dom* gene sequence predicts two major products of the SWI2/SNF2 class of DNA-dependent ATPase, implicating Dom proteins in chromatin modification/nucleosome remodeling (Ruhf *et al.* 2001). Consistent with this idea, Dom protein is associated with the Tip60 acetyltransferase complex and functions in histone exchange (Kusch *et al.* 2004, Lu *et al.* 2007, Börner and Becker 2016); moreover genetic analysis showed that *dom* synergizes with numerous other loci that encode chromatin-associated proteins (Ellis *et al.* 2015). Previous characterizations of *dom* alleles also revealed a repressive role on homeotic genes (Ruhf *et al.* 2001) and E2F targets; the latter indicating that *dom* may function to restrict cell proliferation (Lu *et al.* 2007). Additional functions associated with *dom* include stem cell maintenance and renewal (Xi and Xie 2005, Morillo Prado *et al.* 2013, Yan *et al.* 2014, Börner and Becker 2016) and regulation of telomere capping (Rong 2008). Genetic interaction analyses have also linked *dom* to Notch signaling (Hall *et al.* 2004, Eissenberg *et al.* 2005, Gause *et al.* 2006, Kwon *et al.* 2013). The apparent roles of Dom in both gene repression and activation are predicted by its participation in the Tip60 complex (Gause *et al.* 2006, Schirling *et al.* 2010).

We previously reported a wing phenotype modifier screen designed to expand the gene network contributing to Dom function (Kwon *et al.* 2013). A prominent group of modifiers identified in this screen includes loci that regulate growth, proliferation and autophagy. Notably, we found that multiple genotypes containing down-regulated autophagy loci exhibited enhanced *dom* phenotypes. Given the well-described cross-talk and mutual inhibition between autophagy and cell death (Mariño *et al.* 2014), these results may reflect a predominant role of *dom* in cell viability and restriction of cell death (Braun *et al.* 1997, Ruhf *et al.* 2001). Consistent with this idea, loss of *dom* function in ovaries is associated with germline cell loss and apoptosis (Yan *et al.* 2014), and *dom* can also synergize with other mutations to increase cell death (Ellis *et al.* 2015). Here we have investigated the relationship between loss of *dom* function and apoptotic cell death. Using a set of *UAS*-regulated *dom* RNAi strains and wing *Gal4* drivers we found that targeted expression of *dom* RNAi leads to potent activation of Dcp-1, a marker for induction of apoptosis, as well as depression in the levels of DIAP1, an apoptosis inhibitor. We also observe significant genetic interactions between strains showing *dom* RNAi-mediated phenotypes and strains carrying altered dosages of cell death associated products. Additionally, using a genetic construct that reports the epigenetic state of proapoptotic loci, we determined that loss of *dom* activity leads to derepression of the reporter. Finally, loss of *dom* function was associated with excess cell proliferation, as measured through incorporation of BrdU. These results support roles for *dom* as a pleiotropic regulator, that can block both apoptosis and cell proliferation.

## Materials and Methods

### Drosophila strains

Strains were obtained from the following labs: *C96-GAL4* (G. Boulianne, Toronto), *UAS-Hid* (H. Ryoo, NYU Medical Center), *IRER{ubi-DsRed}* (L. Zhou, University of Florida), *C96-domR* was described previously (Kwon *et al.* 2013).

The following strains were obtained from Bloomington Stock Center (BL# in parentheses):

*w[1118]*; *P{w[+mC]=UAS-rpr.C}14* (5824), w*; *ft^G-rv^* *P{neoFRT}40A/CyO*; *P{UAS-wts.MYC}3/TM6B*, *Tb^1^* (44258), *y[1] v[1]*; *P{y[+t7.7] v[+t1.8]=TRiP.HMC03419}attP40 reaper* (51846), *Df(3L)H99*, *kni[ri-1] p[p]/TM3*, *Sb[1]* (1576), *w[1118]*; *Df(3L)ED225*, *P{w[+mW.Scer\FRT.hs3]=3′.RS5+3.3′}ED225/TM6C*, *cu[1] Sb[1]* (8081), *y[1] v[1]*; *P{y[+t7.7] v[+t1.8]=TRiP.HMS01854}attP2* (*domIR* 38385), y[1] sc[*] v[1]; P{y[+t7.7] v[+t1.8]=TRiP.HMS02208}attP2 (*domIR* 41674), y[1] sc[*] v[1]; *P{y[+t7.7] v[+t1.8]=TRiP.HMS02162}attP2/TM3*, *Sb[1]* (*domIR* 40914), y[1] sc[*] v[1]; *P{y[+t7.7] v[+t1.8]=TRiP.HMC04203}attP2* (*domIR* 55917), *P{w[+mC]=UAS-Dcr-2.D}1*, *w[1118]*; *P{w[+mW.hs]=en2.4-GAL4}e16E*, *P{w[+mC]=UAS-2xEGFP}AH2* (25752), *P{w[+mC]=UAS-Dcr-2.D}1*, *w[1118]*; *P{w[+mW.hs]=GawB}bbg[C96]* (25757), w[*]; *P{w[+mC]=UAS-DIAP1.H}3* (6657), w[*]; *P{w[+mC]=UAS-P35.H}BH1* (5072), y[1] w[*]; *P{w[+mC]=lacW}Diap1[j5C8]/TM3*, *Sb[1]* (12093)

### Genetic interaction tests

The *C96-domR* strain produces a dominant, partially-penetrant wing nicking phenotype that was validated as a *dom* loss-of-function phenotype (Kwon *et al.* 2013). Strains in Table 1 associated with loss or gain of function for cell death loci were each crossed with the *C96-domR* strain. Control crosses include *C96-domR* mated with *w^1118^* flies and *C96-Gal4* mated with *UAS*-regulated and deficiency strains. Genetic interaction, scored as either enhancement or suppression, was measured by changes in the penetrance of wing nicking relative to control crosses that were run simultaneously. In the *C96-domR* x *w^1118^* control crosses described in Table 1 we typically observed a wing nicking penetrance averaging 25%, where wings are scored as positive if they contain one or more anterior margin nicks (Kwon *et al.* 2013). In Table 1 we present the ratio of the percentages of nicked wings (experimental/control class), where a value greater than 1 is enhancement, and less than 1 is suppression. All assays were repeated at least twice, with a minimum of four vials of offspring scored. Significance of the data were calculated utilizing the raw numbers of nicked and un-nicked wings for a Chi square test. Chi square P values shown in Table 1 are uncorrected.

**Table 1:** C96-domR & Genetic Interactions with Cell Death Associated Products

Genotype	Nick Ratio	N	*C96-Gal4* Control	N
*w^1118^*	1.0	>145	NE	1000
*UAS-P35*	0	220	NE	518
*UAS-DIAP*	0.14	350	NE	659
*UAS-Hid*	3.36*^a^*	54	4.5% nicks*^a^*	530
*UAS-Warts*	1.8	449	NE	204
*UAS-Reaper*	Lethal	—	Lethal	—
*UAS-Reaper RNAi*	0.13	186	NE	218
*Df(3L)H99*	0.40	130	NE	155
*Df(3L)ED225*	0.58	104	NE	110

The *C96-domR* strain was tested for phenotypic modification when combined with genotypes carrying gain or loss of function in cell death loci. Tester genotypes were scored as transheterozygotes with the *C96-domR* chromosome. *w^1118^* control crosses were included for each test and used to calculate the nick ratio for each set of crosses. In the table we express the percent of nicked wings as the ratio of experimental %/control %. Ratios greater than 1.0 represent enhancement, and ratios less than 1.0 represent suppression. A minimum of 146 control wings were scored for each *C96-domR* experimental cross. Using a Chi square test all viable crosses produced phenotypic modifications that were highly significant (*P* < 0.001) except for *Df(3L)ED225* (*P* = 0.02) which was significant. Phenotypes were observed in *C96-Gal4* control crosses for only two tester strains. *UAS-Reaper* did not produce viable offspring with either the control or experimental crosses.

a For the control cross *C96-Gal4 x UAS-Hid* we found 4.5% of wings were nicked. This compares with 92.6% nicked wings in the *C96-domR* x *UAS-Hid* experimental cross, and 26.2% nicked wings in the *C96-domR* x *w^1118^* control cross. We corrected the experimental cross value from 92.6 to 88.1% prior to calculating the nick ratio shown in the table.

### Antibody staining of third instar larval wing discs

Imaginal wing discs were dissected in 1X phosphate buffered saline (PBS), fixed for 20 min in 4% paraformaldehyde, and washed 3 times (1X PBS) prior to being permeabilized with 0.3% Triton X-100 in PBS (PBST) for 20 min, and washed once more in 1X PBS (Moberg *et al.* 2005). The discs were then incubated overnight, at 4°, with 10% normal goat serum (NGS) and primary antibody in 0.1% PBST. Subsequently, the discs were washed 5 times (0.1% PBST) and then incubated overnight, at 4°, with NGS and secondary antibody in 0.1% PBST. After the discs were washed 5 more times, they were incubated overnight in n-propyl gallate in glycerol at 4°, and prepared for confocal microscopy. Confocal images were gathered with a Zeiss LSM710 confocal microscope and imaged using the identical optical settings. Images are merged projections. Images were assembled with Photoshop software (Adobe). Primary antibodies include mouse anti-β-Gal (1:1000; Promega); mouse anti-BrdU (1:50; Becton Dickinson); rabbit anti-cleaved Dcp-1 (1:100; Cell Signaling); mouse anti-DIAP1 (1:50; DSHB); rabbit anti-GFP (1:1000; Molecular Probes). The secondary antibodies used are Alexa 647 (1:100) and goat anti-mouse-Cy3 (1:100; Jackson Labs).

### BrdU incorporation assays

Imaginal wing discs were dissected in room temperature Schneider’s medium. Directly following dissection, the discs were transferred into 500μl of Schneider’s medium containing 1X BrdU (3.1 ug/ml), and then incubated at room temperature with gentle agitation for 60 min. Discs were then washed once with room temperature Schneider’s medium, and twice with room temperature 1X PBS, prior to being fixed overnight at 4° in 0.75% paraformaldehyde + 0.01% Tween-20. Subsequently, the discs were washed 5 times in 1X PBS, DNAse treated at 37° for 45 min (20X dilution of RQ1 DNase, Promega), and washed 3 times (0.1% PBST). The discs were then stained with anti-BrdU as described above.

### Mounting of wings

Wings representative of the average severity of wing nicking for each of the strains were mounted onto a slide with Euparol and photographed using a light microscope (Hall *et al.* 2004). The photographs were put in gray scale and sharpened using Adobe Photoshop.

### Reagent and Data Availability

Strains available upon request. The authors affirm that all data necessary for confirming the conclusions of this article are represented fully within the article and its tables and figures.

## Results

### Localized Down regulation of dom in the wing elicits both cell death and hyperproliferation

**Figure 1:**
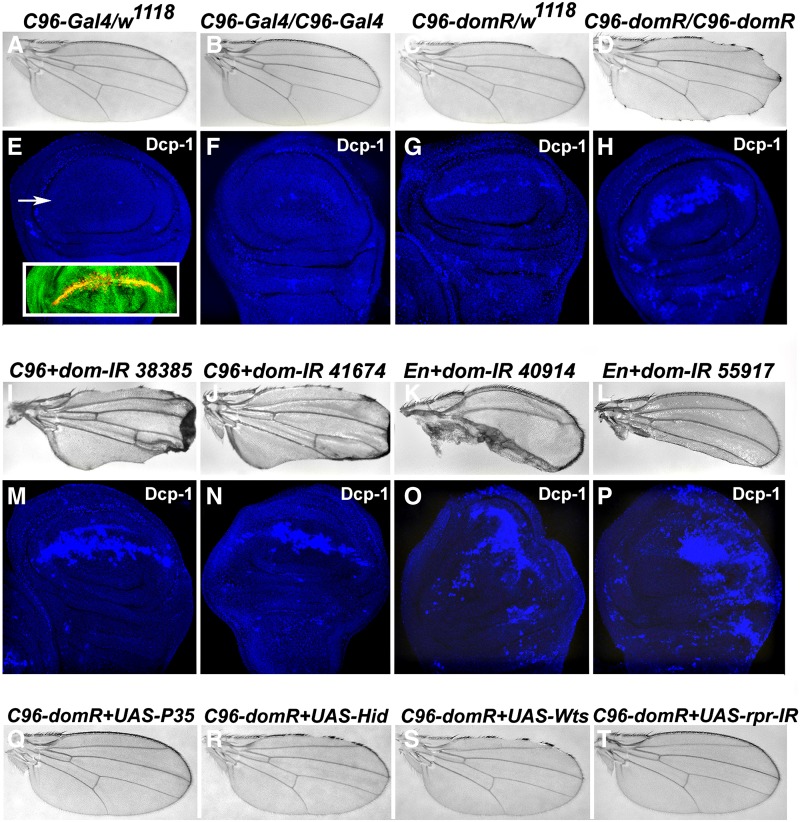
Expression of *dom* RNAi in the wing elicits cell death. Wing mounts were prepared from following strains: *C96-Gal4/w^1118^* and *C96-Gal4/C96-Gal4* (panels A-B); *C96-domR/w^1118^* and *C96-domR/ C96-domR* (panels C-D). Extent of margin loss reflects dose of *C96-domR*. Wing discs from these strains were stained with antibody to cleaved Dcp-1 protein to measure cell death: *C96-Gal4/w^1118^* and *C96-Gal4/C96-Gal4* (panels E-F); *C96-domR/w^1118^* and *C96-domR/ C96-domR* (panels G-H). Cell death levels match wing margin nicking in adult wings. The inset in panel E shows the domain of *C96-Gal4* activity across the dorsal-ventral wing margin (arrow), as reported by yellow color *UAS-GFP*. Four additional *dom TRiP* RNAi constructs were driven by either *C96-Gal4* (panels I, M: BL 38385 and panels J, N: BL 41674) or *En-Gal4* (panels K, O: BL 40914 and panels L, P: BL 55917). Three of these constructs (38385, 41674 and 40914) target both major *dom* A and B form transcripts (Ruhf *et al.* 2001), whereas the 55917 strain targets only the B transcript. The region of adult wing loss again reflects areas undergoing cell death. Panels Q-T show wing mounts from *C96-domR* outcrossed to *UAS-P35* (panel Q), *UAS-Hid* (panel R), *UAS-Warts* (panel S), and a *UAS-TRiP* RNAi strain targeting *Reaper* (panel T). Suppression and enhancement is consistent with the cell death phenotype of *C96-domR* wings (also see Table 1).

The recombinant chromosome strain *C96-domR* contains a wing margin *Gal4* driver (*C96*) and *UAS-RNAi* transgenes directed against a sequence common to all *dom* transcripts. The *C96-domR* chromosome produces a dominant, and partially-penetrant wing nicking phenotype that is enhanced by various *dom* alleles, and suppressed by overexpression of a wild type version of *dom* RNA (Kwon *et al.* 2013). Figure 1 (A-D) shows the wing nicking phenotypes of *C96-domR* heterozygotes and homozygotes along with *C96-Gal4* controls. The homozygous phenotype is severe and completely penetrant, with significant loss of the anterior and posterior wing margins, and some blade material (Figure 1D). Imaginal wing discs from these strains were stained with cleaved Dcp-1 antibody to detect apoptosis. The control strains show occasional areas of staining throughout the disc (Figure 1E-F), whereas *C96-domR* heterozygous and homozygous discs show significantly higher levels of staining across the margin (Figure 1G-H), within the domain of *C96-Gal4* expression (Figure 1E inset and Helms *et al.* 1999). We validated these effects with additional RNAi strains from the Bloomington TRiP collection targeting *dom* sequences in four different regions of the transcripts. When *C96-Gal4* was used to drive these hairpin constructs very strong wing margin defects were produced in heterozygotes (Figure 1I-J); utilizing *En-Gal4* we observed massive loss of posterior wing compartment material in heterozygotes (Figure 1K-L). Wing discs from each of these crosses were stained with cleaved Dcp-1 antibody, revealing high levels of staining in the regions of *Gal4* activity (Figure 1M-P).

We extended these data by testing for genetic modifications of the *C96-domR* heterozygous phenotype through altered dosage of the products of cell death loci. *C96-domR* flies were outcrossed to strains carrying *UAS*-regulated components of the cell death pathway and the wings were scored for penetrance of nicking *vs.* crosses to control *w^1118^* flies (Table 1). We observed that coexpression of inhibitors of apoptosis, P35 and DIAP1, strongly suppressed wing nicks, whereas coexpression of two pathway components, Hid and Warts (Bergmann 2010) enhanced. Furthermore, loss of function for *reaper* via coexpression of *reaper* RNAi led to significant nick suppression, as did chromosomal deletions which eliminate multiple cell death pathway loci (*Df(3L)H99* and *Df*(3L)*ED225*). Representative wings exhibiting enhanced and suppressed *C96-domR* phenotypes are shown in Figure 1 panels Q-T. These results support the contention that *dom* wing phenotypes derive from elevated levels of apoptosis.

**Figure 2:**
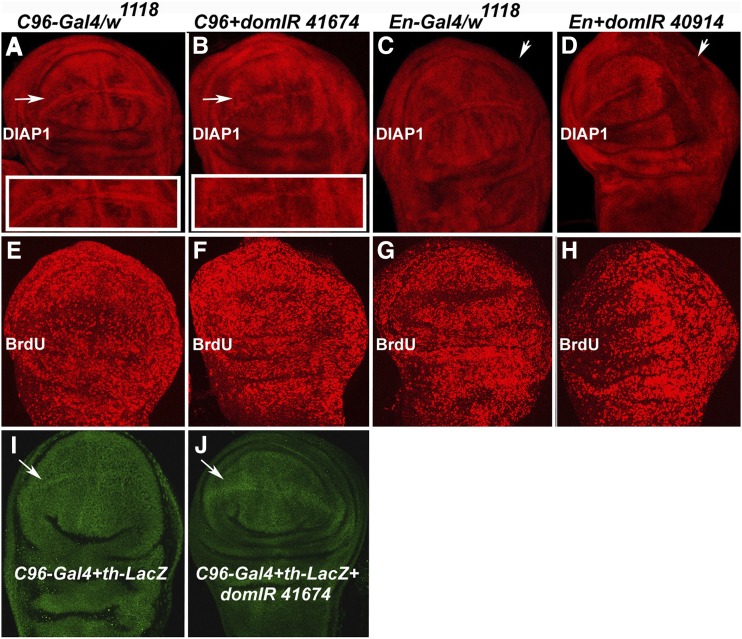
Further effects of *dom* RNAi expression on cell death pathway and proliferation. Wing discs from the following strains were stained with antibody to DIAP1 protein: *C96-Gal4/w^1118^* and *C96-Gal4*/*dom TRiP* RNAi BL 41674 (panels A-B). Arrows in A and B show dorsal-ventral wing margin area that is enlarged in the insets, highlighting diminished stain across the margin in *C96-Gal4*/*dom TRiP* discs. *En-Gal4/w^1118^* and *En-Gal4*/*dom TRiP* RNAi BL 40914 (panels C-D). Arrows in C and D point to posterior compartment of wing disc, the region of *En-Gal4* expression (data not shown). Depression of DIAP1 stain is evident in posterior compartment of wing disc, including the dorsal-ventral margin in *En-Gal4*/*dom TRiP* discs. Wing discs from the same strains described above were also stained for incorporation of BrdU (Moberg *et al.* 2005): *C96-Gal4/w^1118^* and *C96-Gal4*/*dom TRiP* RNAi BL 41674 (panels E-F). We could not detect significant differences in BrdU incorporation between these discs. *En-Gal4/w^1118^* and *En-Gal4*/*dom TRiP* RNAi BL 40914 (panels G-H). Posterior compartment of wing disc oriented rightward, as in panels C and D. Elevation of BrdU incorporation is evident in posterior compartment of wing discs in *En-Gal4*/*dom TRiP* discs, reflecting excess cell proliferation. Wing discs of the genotype *C96-Gal4* + *th-LacZ* (panel I) and *C96-Gal4* + *th-LacZ* + *dom TRiP* RNAi BL 41674 (panel J) were stained with antibodies to β-Gal to monitor activity of the *DIAP1* (*th*) locus. No depression in activity was evident in discs expressing *dom* RNAi (J) *vs.* the control discs (I).

As proapoptotic activity is regulated by activity of the DIAP1 protein (Lee *et al.* 2011), we assayed DIAP1 levels in discs with localized depressions of *dom* function. Normally DIAP1 protein accumulates widely in wing discs with marked accumulation along the dorsoventral margin (Ryoo *et al.* 2002). Figure 2 shows DIAP1 staining in control *C96-Gal4* discs and *C96-Gal4* driving *dom* RNAi. In contrast to the controls, there is a marked reduction of DIAP1 along the wing margin (panels A and B). Moreover, when *En-Gal4* is used to drive *dom* RNAi expression we observe reduced DIAP1 staining within posterior relative to anterior regions of wing discs (Figure 2, panels C-D. Therefore, the elevated levels of apoptosis within regions of discs depressed in *dom* function (Figure 1) is correlated with down regulation of the cell death inhibitor DIAP1. The effect on DIAP1 levels does not appear to be at the level of transcription. Utilizing a *thread-LacZ* reporter (*th-LacZ*) reflecting transcription of the *diap1*/*th* locus, we do not observe lower levels of activity along wing margins expressing *dom* RNAi (Figure 2 I, J).

In a cell culture based screen for regulators of E2F targets, *dom* was identified as an E2F repressor; further, *dom* mutation was found to interact genetically with strains showing excessive or diminished cell proliferation in eye tissue (Lu *et al.* 2007). We investigated the effects of *dom* RNAi expression on cell proliferation, measured through incorporation of BrdU (Moberg *et al.* 2005). When *dom* RNAi was driven by *C96-Gal4* we could not detect significant effects on BrdU incorporation relative to the control discs (Figure 2 E-F). However, *En-Gal4* driving *dom* RNAi led to significant increases in BrdU incorporation within the posterior compartment of the wing disc (Figure 2 G-H). Additionally, hyperproliferation of cells within the posterior compartment of these discs can be manifested as misshapen discs, for example, Figure 2H. Therefore, depression in *dom* function can be correlated with elevated levels of cell proliferation.

### Expression of dom RNAi alters the epigenetic state of IRER near proapoptotic loci

**Figure 3:**
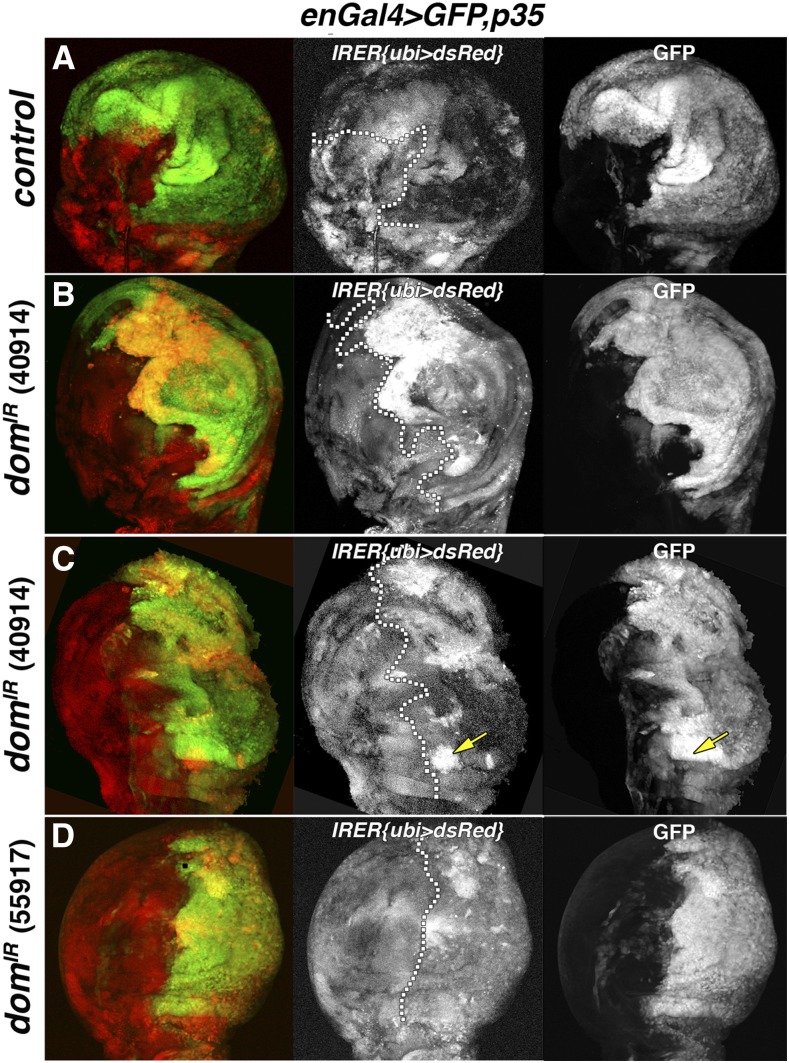
Effect of *dom* RNAi expression on the *IRER* RHG reporter. Dissected 3^rd^ instar wing discs from the following strains were imaged to detect GFP (right panels), RFP (center panels) and merge (left panels): *En-Gal4,UAS-GFP,UAS-P35,IRER{dsRed}* (panel A), *En-Gal4,UAS-GFP,UAS-P35,IRER{dsRed}*+*UAS-dom-RNAi* #40914 (panels B and C), *En-Gal4,UAS-GFP,UAS-P35,IRER{dsRed}*+*UAS-dom-RNAi* #55917 (panel D). Dotted lines divide the anterior (left) and *En-Gal* expressing posterior (right) domains. Arrows in 3C panels indicate a region of intense IRER (red) signal that appears to show only partial overlap with GFP/En signal. However, the GFP only channel validates that this intense IRER signal overlaps entirely with GFP.

Expression of the proapoptotic genes *reaper*, *sickle* and *hid* has been shown to be regulated by an irradiation-responsive enhancer region (IRER) located upstream of *reaper* (Zhang *et al.* 2008). Whereas early embryos have been shown to undergo apoptosis in response to irradiation, later embryos transition to a state that is not responsive. This developmental transition is mediated by epigenetic silencing of the IRER. The state of IRER accessibility can be monitored in the *IRER{ubi-DsRed}* strain (Zhang *et al.* 2015). This strain contains a *ubiquitin-DsRed* reporter that was inserted into IRER via homologous recombination. In this strain, the open or closed chromatin structure of IRER is reflected by the expression of *ubiquitin-DsRed* (Zhang *et al.* 2015). We investigated the effect of *dom* RNAi expression in wing discs on the levels of the *ubiquitin-DsRed* reporter (Figure 3). *UAS-P35* was included in the genotype to prolong the survival of cells that initiate the apoptosis pathway (Hay *et al.* 1994) and thereby preserve the reporter signal. Control discs containing *En-Gal4*, *UAS-GFP*, *UAS-P35* and *IRER{ubi-DsRed}* show variegated, but overall low levels of *ubiquitin-DsRed* activity (Figure 3, panel A). This variegated expression in controls matches the original observations of Zhang *et al.* (2015). Moreover, we found that the misshapen nature of discs associated with *domIR* expression enhanced the irregularity of the *IRER* signal. In contrast, discs containing *En-Gal4* driving *UAS-P35* and either of two *dom* RNAi constructs in a *IRER{ubi-DsRed}* genetic background exhibit high levels of *IRER{ubi-DsRed}* activity in the GFP-positive posterior compartment: the region of *En-Gal4* expression (Figure 3, panels B-D). Therefore, depression of *dom* function appears to modify the chromatin structure proximal to proapoptotic genes, potentially allowing higher levels of expression.

## Discussion

The initial characterization of a *dom* mutation implicated the locus in cell death regulation as homozygous *dom* larvae contained necrotic lymph glands and a deficiency in hemocytes (Braun *et al.* 1997). Loss of *dom* function subsequently was associated with apoptosis in the germline (Yan *et al.* 2014). Here we have shown that RNAi-mediated depressions in *dom* function lead to activation of apoptosis in the wing disc, as measured through staining for cleaved Dcp-1 and resultant adult wing phenotypes (Figure 1). Current models of cell death regulation propose that DIAP1 binds and inhibits the activity of the cleaved effector Caspase Dcp-1. This inhibition appears to be overcome by elevated levels of the RHG proteins (Reaper, Hid and Grim), which bind DIAP1 and lead to its degradation (Bergmann 2010). Consistent with these models, the *dom* wing phenotype is sensitive to the dosage of several proapoptotic gene products as well as the inhibitors P35 (Hay *et al.* 1994) and DIAP1 (Table 1). Further, we found that *dom* RNAi expression leads to depression in the levels of DIAP1 protein, without a detectable effect on the levels of *diap1* gene (*th*) transcription, measured with a *th-LacZ* reporter (Figure 2). A prediction of this model, not yet tested, is that *dom* IR-induced depressions in DIAP1 levels would be suppressed via loss of function for RHG loci.

Given these data, along with the classic description of *dom* as a genetic repressor (Ruhf *et al.* 2001, Lu *et al.* 2007) a reasonable explanation for the effects of *dom* RNAi invokes derepression of RHG loci. To address this possibility, we assayed the expression of a *ubiquitin-DsRed* reporter, resident within an irradiation-responsive enhancer region (IRER) of the proapoptotic RHG loci (Zhang *et al.* 2008). We found that *dom* RNAi expression in wing discs strongly increased the level of expression of *ubiquitin-DsRed* relative to control discs (Figure 3). Therefore, the loss of *dom* function likely alters the chromatin state surrounding the RHG loci, leading to their elevated expression. The consequent increase in amounts of RHG proteins would likely launch apoptosis.

Dom has also been linked to regulation of cell proliferation. Lu *et al.* (2007) found that Dom associates with E2F at promoters and contributes to a repressed state at loci involved in cell proliferation. Genetic interaction studies also implicate *dom* in proliferation (Lu *et al.* 2007, Kwon *et al.* 2013, Ellis *et al.* 2015). We tested the prediction that loss of *dom* function can lead to excess proliferation by measuring BrdU incorporation. We observed that *En-Gal4* directed expression of *dom* RNAi in the posterior compartment of wing discs led to higher levels of BrdU incorporation (Figure 2). The increased BrdU levels were also associated with enlarged and misshapen posterior regions of the discs, presumably due to the excess number of cells. The adult phenotype derived from wing discs of this genotype shows massive loss of posterior compartment material (Figure 1), indicating that cell death ultimately masks the hyperproliferation effect. Importantly, these data do not necessarily implicate *dom* directly in genetic regulation of hyperproliferation. There are multiple lines of evidence linking apoptosis to a compensatory proliferation response in damaged tissues (Fogarty and Bergmann 2017). In any case, the phenotype derived from loss of *dom* function is pleiotropic, consistent with its broad range of genetic interactions with other regulatory proteins (Ellis *et al.* 2015).
